# The biomechanical coordination during oropharyngeal swallowing: an evaluation with a non-invasive sensing system

**DOI:** 10.1038/s41598-017-15243-6

**Published:** 2017-11-09

**Authors:** Qiang Li, Yoshitomo Minagi, Takahiro Ono, Yongjin Chen, Kazuhiro Hori, Shigehiro Fujiwara, Yoshinobu Maeda

**Affiliations:** 1 State Key Laboratory of Military Stomatology and National Clinical Research Center for Oral Diseases and Shaanxi International Joint Research Center for Oral Diseases, Department of General Dentistry & Emergency, School of Stomatology, The Fourth Military Medical University, Xi’an, 710032 China; 2 Department of Prosthodontics, Gerodontology and Oral Rehabilitation, Osaka University Graduate School of Dentistry, Suita, 565-0871 Japan; 3 Division of Comprehensive Prosthodontics, Niigata University Graduate School of Medical and Dental Sciences, Niigata, 951-8514 Japan

## Abstract

Swallowing is a very important and complex physiological behaviour. The dynamic of swallowing has created great interest as any procedural abnormality will result in dysphagia and even lower quality of life. However, a non-invasive evaluation of biomechanical coordination during oropharyngeal swallowing, which includes the activities of the tongue, the hyoid and swallowing-related muscles, has not yet been achieved. In the present study, we recruited fifteen subjects, and a non-invasive sensing system composed of a pressure sensor, a bend sensor, surface electrodes and a microphone was created to simultaneously monitor tongue pressure, hyoid motion, and surface EMG of swallowing-related muscles, as well as take sound recordings, when the subjects swallowed 5 ml of water. In addition to obtaining the durations of certain motor events, the considerable time (beginning, peak and ending time) of tongue pressure production, suprahyoid and infrahyoid muscle activity and hyoid motion were successfully measured. Moreover, the significant correlations between swallowing-related muscles, tongue pressure, and the hyoid were confirmed. These findings suggest that the non-invasive sensing system has potential as a good candidate for monitoring and evaluating the oropharyngeal process of swallowing, which may be useful in clinical work involving dysphagia evaluation and rehabilitation.

## Introduction

Swallowing is a complicated physiological process involving the recruitment of several structures in a very short time to transport a bolus from the mouth to the stomach. It plays a crucial role in food transmission, nutrition intake, physical growth and even human survival. This process is subdivided into the oral phase, the pharyngeal phase and the oesophageal phase from an anatomical point of view^1^. Any abnormality in the process (i.e., dysphagia) would not only cause dehydration, malnutrition, weight loss, aspiration and pneumonia but could also negatively impact daily activities and quality of life^2^.

Although the oral and pharyngeal phases of swallowing are presented sequentially, the physiologic reality is that both of the phases are integrally related^3,4^. Physiologically, the transmission of the bolus through the oral and pharyngeal cavities involves a series of systematic biomechanical motor events. However, until now, the complexity of these events and the difficulty of monitoring these structural actions are undoubtedly the major reasons for our relative lack of knowledge about the motor mechanisms involved in oropharyngeal swallowing. Therefore, in modern society, it is the pursuit of the clinician to monitor oropharyngeal swallowing behaviour and evaluate the related functions conveniently and noninvasively for the timely diagnosis of any swallowing defects.

During the oropharyngeal process of swallowing, the tongue participates in collecting and positioning the food bolus^5^ and plays a critical role in the posterior propulsion of the bolus with the help of tongue pressure arising from its contact against the hard palate^6^. In addition, the hyoid elevation towards the base of tongue facilitates the upper oesophageal sphincter (UES) opening^7^, consequently allowing the food pass through the pharynx to the oesophagus. Obviously, optimal oropharyngeal swallow performance requires the intricate events of the tongue and the hyoid to occur in concert with each other to transport the bolus safely and efficiently.

The fine-tuned relationship between the tongue and the hyoid, which are the representative anatomical structures of the oral cavity and the larynx, could not exist without their muscular connection. Various literature has documented that the suprahyoid (SH) muscles and the infrahyoid (IH) muscles are not only involved in the oral phase, contributing to fixing and elevating the tongue^8^, but are also involved in the pharyngeal phase when the hyolaryngeal complex elevates and returns to rest^9,10^. Accordingly, electromyography (EMG) of certain muscles could be a useful parameter for speculating the oropharyngeal function, and the temporal actions of the tongue body and the hyoid has ever been indirectly reflected by measuring the EMG of the oral and laryngeal-related muscles^11^. Moreover, previous studies have supplied the kinetic correlation between submental muscle activity and the hyoid by concurrent application of the submental surface EMG (sEMG) and a movement transducer on the anterior neck over the larynx^12^ or sEMG and videofluorography (VF)^13^. Recently, researchers took advantage of the EMG bioimpedance (EMBI) system and successfully mapped characteristic functional changes in the pharynx during swallowing, specifically laryngeal elevation^14^. This information indeed deepened our understanding of swallowing.

Currently, there is still a lack of data available that thoroughly describes the biomechanical coordination of tongue pressure generation and hyoid activity in reference to the sEMG of SH and IH muscles during oropharyngeal swallowing. Moreover, although various instruments such as VF, endoscopy, CT, ultrasound, MRI, and so on have been used to monitor the swallowing behaviour in human beings in the past several decades^8,15–18^, certain drawbacks including radiation, expensiveness, inconvenience, and specialization of the aforementioned appliances still limit their popularization and application. The need for the development of non-invasive methods of quantification and visual evaluation of swallowing behaviour or swallowing disorders is growing within the area of odynophagia and dysphagia management.

Therefore, in this investigation, we innovatively took advantage of several non-invasive appliances simultaneously to measure the tongue pressure production, hyoid movement, and SH and IH EMG during normal oropharyngeal swallowing. We intended to use the sensing system to (1) characterise the temporal pattern of the tongue, the hyoid, and the SH and IH muscles, and (2) determine how these oropharyngeal patterns of the tongue and the hyoid are related to and coordinated with the muscle activity. All of our present efforts will shed light on the biomechanical coordination during oropharyngeal swallowing. Furthermore, it could be beneficial for clinicians to simply and efficiently evaluate oropharyngeal swallowing and provide an early diagnosis of dysphagia chair-side and bed-side.

## Materials and Methods

### Subjects

Fifteen adult male subjects with an average age of 27.7 years (range = 25–32 years) without any signs of severe malocclusion, mastication or swallowing problems, neurological disease, structural disorders or other oropharyngeal problems participated in this study. As for the subject number in the current study, we used the sample size software NCSS-PASS 11.0 (NCSS LLT, Utah, USA) to calculate. A study^19^ reported that hyoid movement lagged the onset of tongue-palate pressures in 63.2% of swallows. This phenomenon in our study is 94.7%. So the difference is 31.5%. We used the Tests for one proportion (Non-Zero Null Hypothesis) [Differences] and calculated the sample size as fifteen after setting the power (1-Beta) 0.90, Alpha 0.05, PB (Baseline Proportion) 0.632, d0 (Superiority Difference) 0, and d1 (Actual Difference) 0.315. Informed consent was obtained from each participant after receiving information about the experimental procedure. The study protocol was approved by the Ethics Committee of Osaka University Graduate School of Dentistry (No. H21-E32) and the experiments were performed in accordance with the Declaration of Helsinki (2008) for humans.

### Measuring system and procedure

**Figure 1:**
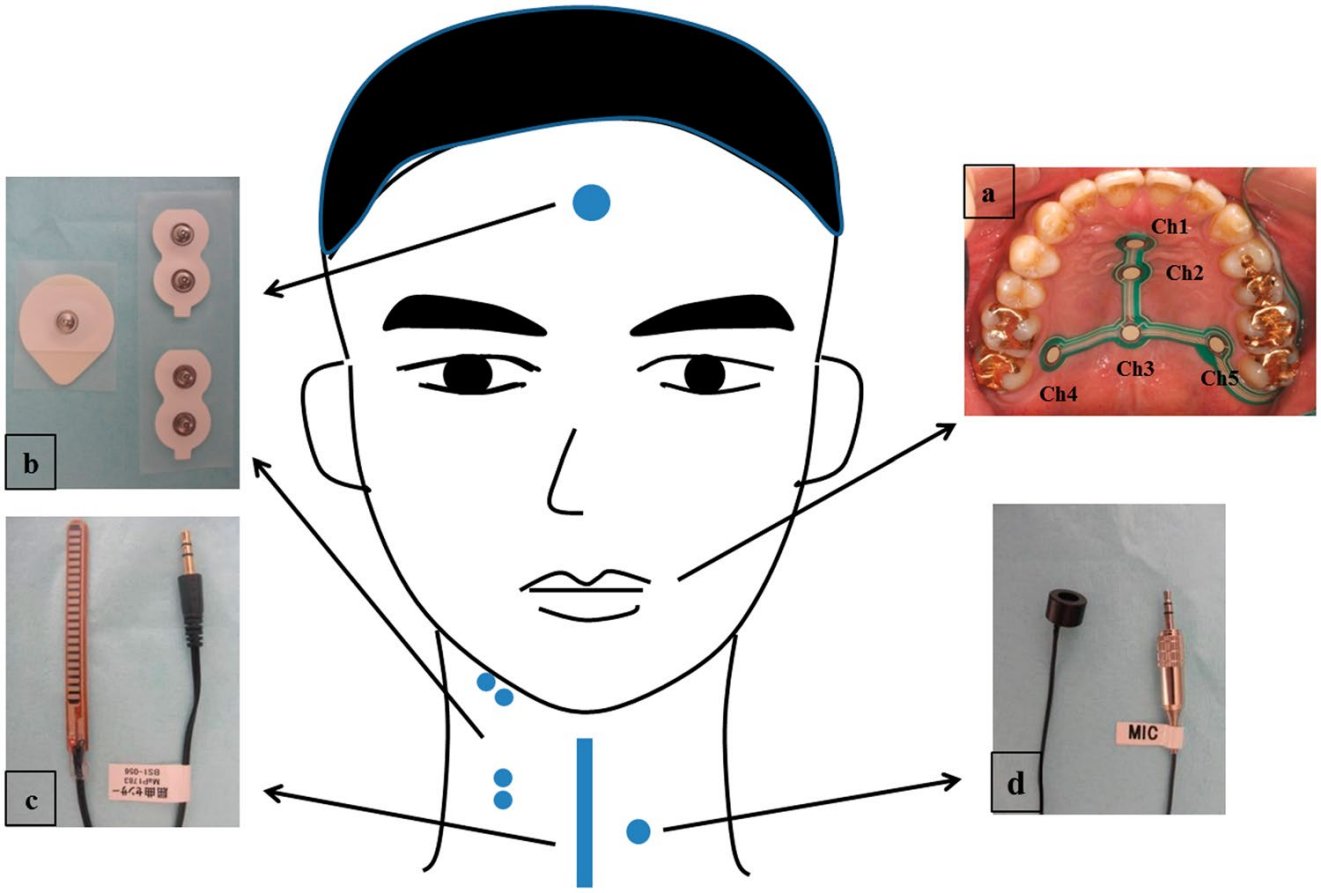
Schematic representations of the sensing system and experimental set-up. (**a**) A subject with tongue pressure sensor sheet, surface electrodes, bend sensor and microphone. (**b**) Tongue pressure sensor sheet. (**c**) Surface electrodes. (**d**) Bend sensor. (**e**) Microphone.

The sensing system consists of four non-invasive devices including a tongue pressure sensor sheet (Fig. 1a), surface electrodes (Fig. 1b), bend sensor (Fig. 1c) and a microphone (Fig. 1d). The tongue pressure produced in the midline and the posterior-lateral part of the hard palate was recorded by the tongue pressure sensor sheet (100 Hz, Nitta, Osaka, Japan) with a thickness of 0.1 mm and 5 measuring points (Ch.1–5). Specifically, Channel 1 to Channel 3 (Ch.1-Ch.3) were placed along the median line anteroposteriorly, and Channel 4 and Channel 5 (Ch.4 and Ch.5) were situated in the posterior–circumferential parts of the hard palate. The sensor sheet was attached to the hard palate by a sheet-type denture adhesive (Touch Correct II, Shionogi, Tokyo, Japan) after the suitable selection from three sizes according to the participant’s palate form^20,21^. We calibrated the sensor sheet by applying negative pressure using a vacuum pump through an air duct in the sensor sheet cable before measurement.

For the EMG recording of the suprahyoid and infrahyoid muscle groups (SH EMG and IH EMG), five surface electrodes (Duo-trode, Myotronics, MA, USA) were utilized for each subject. The subject’s skin was scrubbed to attach the surface electrodes. Since no side-to-side difference was found in the EMG of muscles involved in swallowing in healthy subjects^22^, one pair of electrodes (d = 8 mm, interelectrode distance = 2 cm) was attached to the skin of the right side of the anterior belly of the digastric muscle to measure the EMG of the suprahyoid muscle group. Another pair of electrodes (d = 8 mm, interelectrode distance = 2 cm) was attached to the right side of sternohyoid muscle to measure the EMG of the infrahyoid muscle group. A reference electrode affixed to the forehead served as the ground. Signals from the EMG electrodes were band-passed filtered (100 Hz-10 kHz), amplified (BA1104, Nihon Kohden, Tokyo, Japan), full-wave rectified, and then stored on a computer through an interface (PCI-3133A, Nihon Santeku, Osaka, Japan) at a sampling rate of 10 kHz.

To record the hyoid activity, the bend sensor (73.7 mm × 6.4 mm × 1.0 mm, 1000 Hz, MaP 1783BS1-056, Nihon Santeku Co. Ltd., Japan) was fixed along the midline of the frontal neck with its tip at the level of the prominence of thyroid cartilage when reaching the highest position during swallowing. It could flex physically with the laryngeal motion, and the hyoid activity could be retrieved non-invasively from the produced signal waveform^23^.

Because the symmetry of the swallowing sound could be acquired bilaterally^24^, we placed a microphone (JM-0116, Ono-Sokki, Tokyo, Japan) over the left lateral border of trachea immediately inferior to the cricoid cartilage to detect the timing of bolus passage through the entrance of oesophagus^25^.

The subjects examined in this study were instructed to sit in an upright position with their heads supported by a headrest to avoid head retroflexion and to keep the Frankfort plane horizontal with their feet touching the floor. Then, 5 ml of water (37 °C) was given via syringe and held on the mouth floor until swallowed wholly, one time, upon verbal command. The participant was asked to relax the tongue immediately after each trial. Three repetitions were performed for each subject. The recorded tongue pressure and swallowing sound data were subsequently integrated on a personal computer through an interface board (PCD 100 A, Kyowa Electric Instruments, Tokyo, Japan). The EMG data and the obtained signal from the bend sensor were amplified and stored on the personal computer through a separate interface board (PCI-3133A, Nihon Santeku, Osaka, Japan). To ensure that all of the subjects felt comfortable and that all the devices worked properly, at least one successful practice swallow was completed before recording the experimental data. To synchronize all the data, the trigger signal to start measurement from the swallow scan was sent to the interface board (PCI-3133A, Nihon Santeku, Osaka, Japan); then, tongue pressure, EMG, hyoid motion and swallowing sound were measured at the same time.

### Data analysis

**Figure 2:**
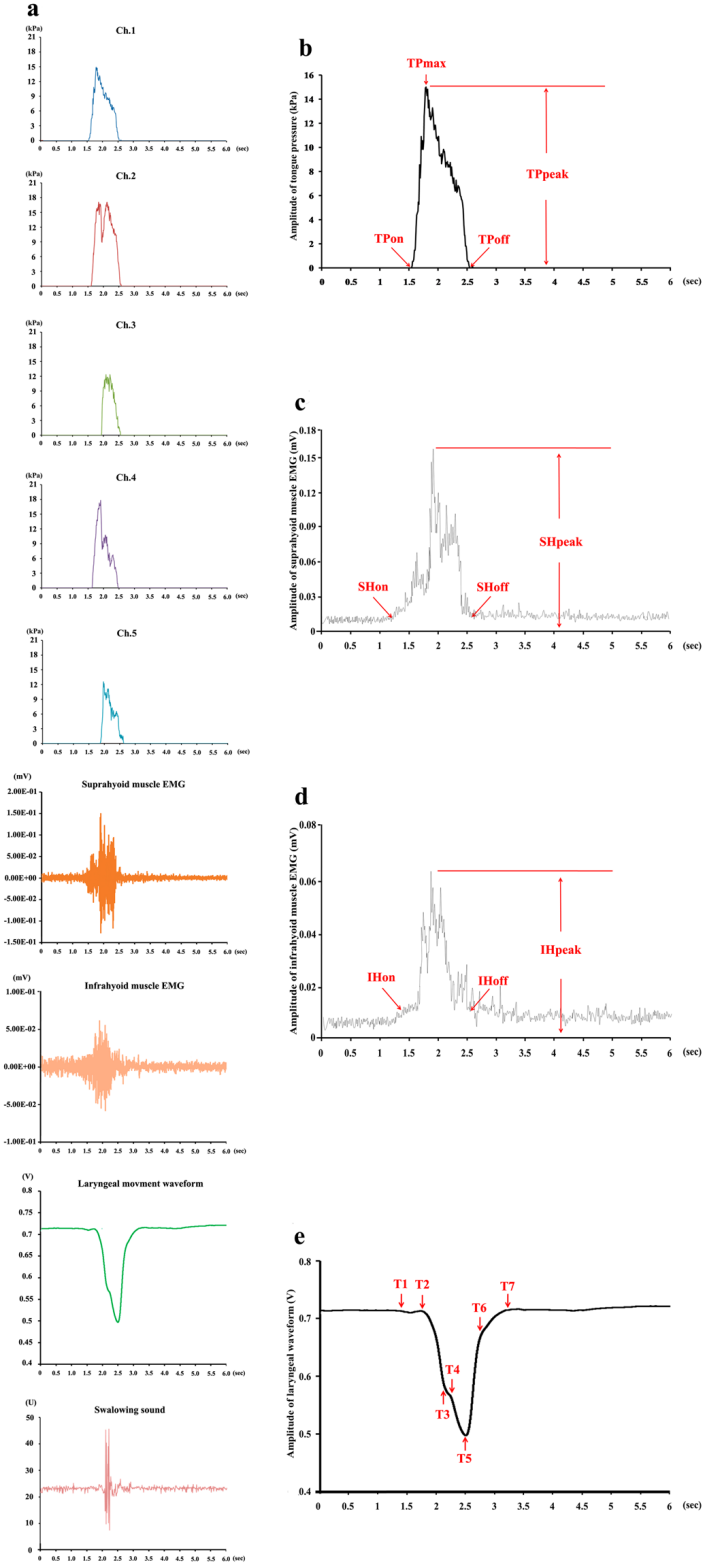
Representative recordings of the noninvasive sensors. (**a**) Decomposed graph of waves of tongue pressure, EMG, laryngeal movement and swallowing sound. (**b**) The data analysis of TP_on_, TP_max_, TP_off_ and DTP. (**c**) The data analysis of SH_on_, SH_off_, SH_peak_ and DSH. (**d**) The data analysis of IH_on_, IH_off_, IH_peak_ and DIH. (**e**) Laryngeal signal waveform and marked time point.

Figure 2a shows the representative raw waves of tongue pressure, EMG of suprahyoid and infrahyoid muscles, laryngeal movement and swallowing sound. The following parameters of tongue pressure on each sensor were recorded (Fig. 2b): time of tongue pressure onset (TP_on_), time of maximum tongue pressure (TPmax), time of tongue pressure offset (TPoff), peak value of tongue pressure (TPpeak) and duration of tongue pressure production (DTP). EMG bursts were full-wave rectified and smoothed (time constant, 20 ms) using the application (MaP1038A, Nihon Santeku, Osaka Japan). The onset, offset, peak value and duration of suprahyoid muscle activity (SHon, SHoff, SHpeak and DSH) and also infrahyoid muscle activity (IH_on_, IH_off_, IH_peak_ and DIH) were measured as the EMG parameters (Fig. 2c,d). The onset time of each EMG burst was the time at which it was beyond 2 standard deviations (SDs) of baseline activity, and the offset time was the time at which it was below 2 SDs^22^. Additionally, we recorded certain time points on the laryngeal movement waveform produced by the bend sensor, i.e., T1, T2, T4, T5 and T6, to represent the onset of slight movement of the hyoid, the onset of rapid movement of the hyoid, the onset of the stationary phase of the hyoid, the offset of the stationary phase of the hyoid, and the offset of movement of the hyoid, respectively (except for T3 and T7, which were confirmed to be meaningless for hyoid activity) (Fig. 2e)^23^. Additionally, the times for T1-T5 and T2-T5 were measured because they could represent the duration from the onset of slight movement of the hyoid to the offset of the stationary phase of the hyoid and the onset of rapid movement of the hyoid to the offset of the stationary phase of the hyoid, respectively. With respect to the swallowing sound, because more than one spike was typically observed in the sound data, the spike with the greatest amplitude was chosen to be the reference time^21^ for comparing the temporal sequence of biomechanical events during oropharyngeal swallowing.

### Statistics

All the data from 45 trials (15 subjects × 3 trials) were analyzed with SPSS 16.0 software (SPSS Inc., Chicago, IL, USA). A one-way analysis of variance (ANOVA) was used to compare the durations of certain physiological activities. To evaluate the sequential order of tongue pressure, muscle EMG and hyoid motion, the uniformity of variance was determined by the Kolmogorov-Smirnov test first. When uniform variance was found, significant differences were determined by repeated-measures ANOVA, and comparison testing was performed with the Bonferroni post hoc test. The interclass correlation coefficient was used to evaluate the correlations between the swallowing-related muscle activity (SH_on_, IH_on_, SH_off_ and IH_off_) and certain biomechanical events of tongue pressure (TP_on_ of Ch.1–5 and TP_off_ of Ch.1–5) and hyoid activity (T1, T2 and T5). All the data were expressed as mean ± S.D. Statistical significance was set at p < 0.05.

## Results

### Duration of biomechanical events during oropharyngeal swallowing

**Figure 3:**
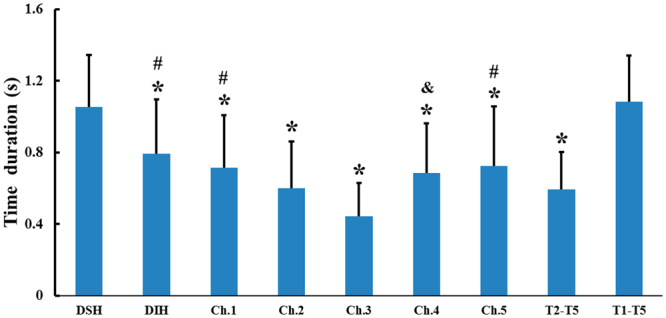
Duration of biomechanical events during oropharyngeal swallowing. DSH, duration of suprahyoid muscle activity; DIH, duration of infrahyoid muscle activity. *p < 0.001 v.s. DSH and T1-T5; ^#^p < 0.001 v.s. TP of Ch.3; ^&^p < 0.005 v.s. DTP of Ch.3.

As shown in Fig. 3, the DSH, DIH, DTP for Ch. 1, Ch.2, Ch. 3, Ch.4, Ch. 5 and the times for T2-T5 and T1-T5 were 1.05 ± 0.29 s, 0.79 ± 0.31 s, 0.71 ± 0.30 s, 0.60 ± 0.26 s, 0.44 ± 0.19 s, 0.69 ± 0.28 s, 0.73 ± 0.33 s, 0.59 ± 0.21 s and 1.08 ± 0.26 s, respectively. The time is longer for DSH when compared with DIH, DTP for Ch.1, Ch.2, Ch.3, Ch.4, Ch.5 and T2-T5 (all p < 0.001). Similar results were also found between T1-T5 and DIH, DTP for Ch.1, Ch.4, Ch.5 and T2-T5 (all p < 0.001). The DTP for Ch.3 shows the shortest duration. The significances were confirmed between the DTP for Ch.3 and the other events (all p < 0.005) except for DTP of Ch.2 and T2-T5 (p > 0.05). In addition, there were no significant differences in the durations between DSH and T1-T5 (p > 0.05), between the DTP for Ch.3 and T2-T5 or among DIH and the DTP for Ch.1, Ch.4 and Ch.5 (all p > 0.05).

### Temporal sequence of biomechanical events during oropharyngeal swallowing

**Figure 4:**
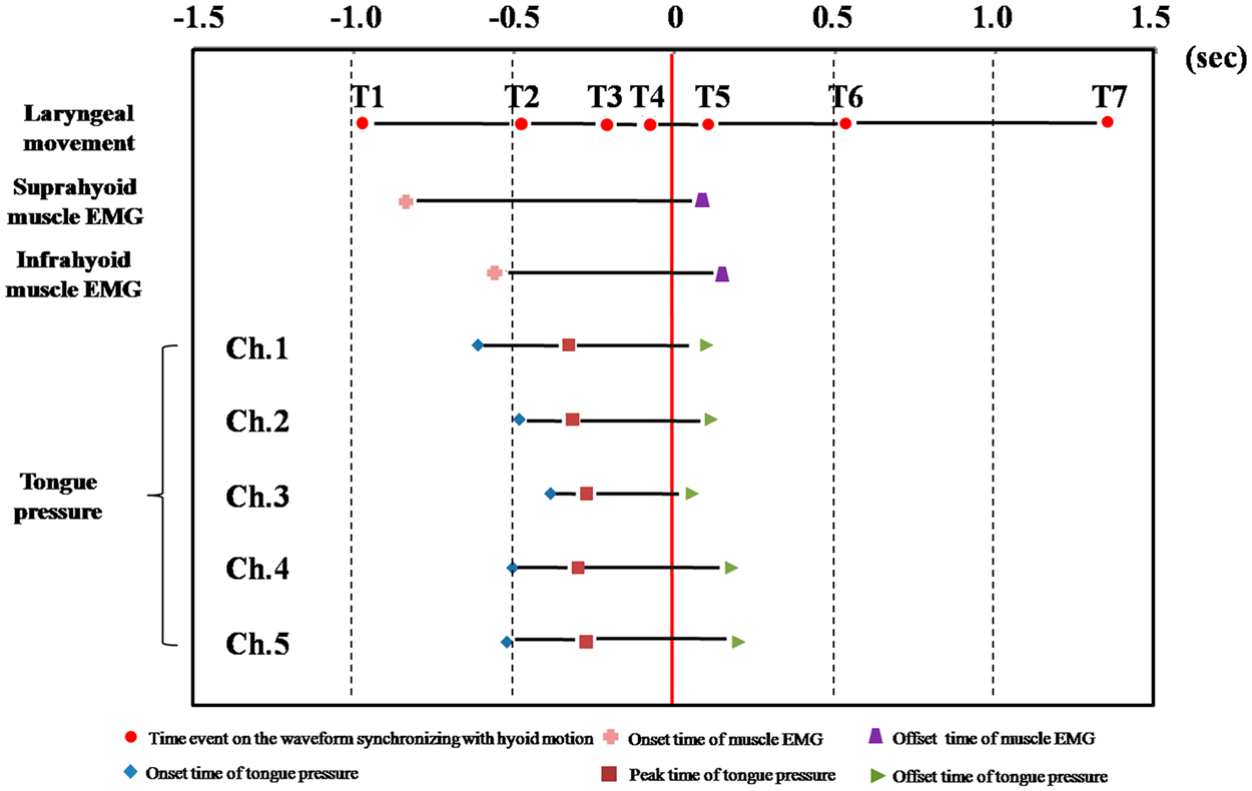
Temporal sequence of biomechanical events during oropharyngeal swallowing. The red line is the swallowing sound that was chosen to be the reference time.

As shown in Fig. 4, the slight movement of the hyoid (T1, −0.97 ± 0.27 s) occurred first among all of the monitored biomechanical events and most closely to the subsequent SH_on_ (−0.83 ± 0.25 s) (p > 0.05). Then, the TP_on_ of Ch.1 (−0.61 ± 0.21 s) appeared with the simultaneous appearances of IH_on_ (−0.55 ± 0.24 s), TP_on_ of Ch.5 (−0.52 ± 0.22 s) and Ch.4 (−0.51 ± 0.19 s), as well as upward movement of the hyoid (T2, −0.48 ± 0.28 s) (all p > 0.05), then followed by the TP_on_ of Ch.2 (−0.49 ± 0.21 s) and Ch.3 (−0.39 ± 0.19 s) (p = 0.11, p < 0.001, respectively). Though the TP_max_ (Ch.1, −0.33 ± 0.25 s; Ch.2, −0.32 ± 0.22 s; Ch.3, −0.27 ± 0.21 s; Ch.4, −0.30 ± 0.22 s; Ch.5, −0.28 ± 0.19 s) occurred before the onset of the stationary phase of the hyoid (T4, −0.08 ± 0.21 s) during swallowing, no significant differences were found among them (all p > 0.05). In addition, the biomechanical events of the offset of the stationary phase of the hyoid (T5,0.12 ± 0.17 s), SH_off_ (0.10 ± 0.28 s), IH_off_ (0.16 ± 0.23) and TP_off_ (Ch.1, 0.10 ± 0.23 s, Ch.2, 0.12 ± 0.24 s; Ch.3, 0.06 ± 0.19 s; Ch.4, 0.18 ± 0.25 s, Ch.5,0.20 ± 0.22 s) appeared without any significant time lags (all p > 0.05).

### Correlation coefficient of biomechanical events during oropharyngeal swallowing

As shown in Table 1, the positive correlations between SH_on_ and T1, SH_on_ and T2, as well as IH_on_ and T2 were noted with moderate correlation coefficients (r = 0.658, p = 0.002; r = 0.616, p = 0.008; r = 0.666, p = 0.005, respectively). In addition, there were significant positive correlations between SH_off_ and TP_off_ at Chs. 1-5 and T5 with moderate correlation coefficients (r = 0.653 p = 0.002; r = 0.594, p = 0.019; r = 0.626, p = 0.008; r = 0.633, p = 0.007; r = 0.613, p = 0.010; r = 0.694, p = 0.001, respectively). This was also the case between IH_off_ and TP_off_ at Chs. 1-5 and T5 (r = 0.656, p = 0.002; r = 0.643 p = 0.003; r = 0.640, p = 0.004; r = 0.580, p = 0.026; r = 0.689, p = 0.001; r = 0.602, p = 0.010, respectively).

**Table 1:** Correlation coefficient of biomechanical events during oropharyngeal swallowing.

Events of muscle EMG	Eventsof the tongue pressure and hyoid activity	*r*	*p*
SH_on_	Ch.1 TP_on_	0.472	0.058
	Ch.2 TP_on_	0.434	0.064
	Ch.3 TP_on_	0.415	0.070
	Ch.4 TP_on_	0.377	0.112
	Ch.5 TP_on_	0.192	0.206
	T1	0.658	0.002
	T2	0.616	0.008
IH_on_	Ch.1 TP_on_	0.543	0.025
	Ch.2 TP_on_	0.302	0.152
	Ch.3 TP_on_	0.277	0.175
	Ch.4 TP_on_	0.252	0.199
	Ch.5 TP_on_	0.198	0.251
	T1	0.258	0.193
	T2	0.666	0.005
SH_off_	Ch.1 TP_off_	0.653	0.002
	Ch.2 TP_off_	0.594	0.019
	Ch.3 TP_off_	0.626	0.008
	Ch.4 TP_off_	0.633	0.007
	Ch.5 TP_off_	0.613	0.010
	T5	0.694	0.001
IH_off_	Ch.1 TP_off_	0.656	0.002
	Ch.2 TP_off_	0.643	0.003
	Ch.3 TP_off_	0.640	0.004
	Ch.4 TP_off_	0.580	0.026
	Ch.5 TP_off_	0.689	0.001
	T5	0.602	0.010

## Discussion

In the present study, a pressure sensor was placed in the oral cavity to measure tongue pressure generated from the contact between the tongue and the hard palate^20^. In addition, a bend sensor was attached to the midline skin of the neck to reflect hyoid motion with its recorded waveform^21,23^, and the surface EMG was conducted to explore the activities of swallowing-related muscles^26^. Furthermore, a microphone that could detect the sound when a bolus passes through the entrance of the oesophagus was used for the sound recordings^25^. With these noninvasive appliances, we built a sensing system that successfully and synchronously measured certain essential oropharyngeal swallowing events without causing any discomfort to the subjects or disturbing their swallowing behaviour. The motor pattern and the temporal coordination of these representative oropharyngeal events were also adequately clarified.

We compared the durations of biomechanical events during oropharyngeal swallowing and observed similar persistent periods of suprahyoid muscle activity and hyoid motion (T1-T5). This is consistent with previous studies that reported the movement synchronization of the suprahyoid muscle and the hyoid used EMG and VF^27^ or EMG and CT^28^. Therefore, the data indirectly indicate the sensitivity and the accuracy of the bend sensor to reflect the hyoid motion. Combined with the aforementioned reports, we consider the suprahyoid muscle as the main driving force of hyoid activity during swallowing. Although there was no difference between the EMG peak value of the suprahyoid muscle (0.040 ± 0.010 mV) and the infrahyoid muscle (0.037 ± 0.017 mV) in the current study, the longer duration of suprahyoid muscle activity than that of the infrahyoid muscle was observed. This phenomenon may arise from the potential and function of the two muscles during swallowing. Additionally, the durations of tongue pressure in the anterior and lateral parts corresponded well with those reported by Hori *et al*.^29^ and Tamine *et al*.^30^.

As for the sensor signals obtained by the sensing system, we could observe certain rules. The tongue pressure signal peaked quickly, and then decreased gradually before disappearing almost simultaneously at each measured part of the hard palate. In addition, the EMG of swallowing-related muscles exhibited similar pattern with quick rise and slow descent. The sensor recorded hyoid activity produced a regular “V”-shaped waveform, with a preliminary movement at the beginning represents the onset of slight movement of the hyoid (T1), followed by rapid downward movement represents the onset of rapid movement of the hyoid (T2) with a subsequent small and obvious notch represents the onset of the stationary phase of the hyoid (T4) until reaching its peak point represents the offset of the stationary phase of the hyoid (T5). Then, the waveform reversed, quickly at first and then slowly after a turning point represents the offset of movement of the hyoid (T6). Finally, it returned to the baseline (T7)^23^. From the view of the initial rapid motion and succeeding slow motion of the monitored organs, we could speculate that more effortful work is needed for triggering the oropharyngeal swallowing, and the later movements is mainly for maintaining swallow smoothly.

With regard to the motor pattern of these representative oropharyngeal events, we noted that the slight movement of the hyoid (T1) was the first monitored biomechanical event during normal swallowing and that it even occurred a little earlier than the SH_on_, but without any significance of time lag. Because the subject in the present study needed to dip the water from the floor of the mouth to the supra-lingual location after the verbal command of swallowing^31^, the precise activity of the tongue tip may contribute to the subtle motion of the hyoid via tongue extrinsic musculature that connects them^32^. Taniguchi and his colleagues^4^ have documented the suprahyoid muscle EMG burst prior to the motion of the anterior and posterior tongue. Our findings not only support this report but also confirm the fact that SH_on_ preceded the lateral tongue activity, i.e., the burst of the suprahyoid muscle was earlier than that of tongue pressure. Once the tongue pressure is produced because of the tongue-hard palate approximation, the hyoid needs to elevate superior-anteriorly to facilitate the subsequent laryngeal vestibule closure, epiglottis reversion and UES opening for the upcoming food bolus^33–35^. Therefore, it is reasonable to observe the simultaneous appearances of tongue pressure and upward movement of the hyoid (T2). Meanwhile, the infrahyoid muscle activity occurred at this time. Palmer *et al*. previously confirmed that little infrahyoid muscle activity occurs when each swallow starts until the hyoid moves sharply^8^. Although the tongue pressure peak value arose before the onset of the stationary phase of the hyoid (T4) during swallowing, significant differences were not found. This suggests that, in the process of oropharyngeal swallowing, powerful tongue-hard palate contact is essential for anchoring the hyoid on the one hand, but on the other hand, the hyoid should stay at its highest position to open the pharyngeal cavity to the greatest extent accordingly in favour of receiving the bolus that is passing the fauces when the tongue pressure reaches its maximum^5^. With the sensing system, we also observed the concurrent offset of the stationary phase of the hyoid (T5), EMG of swallowing-related muscles and tongue pressure. Physiologically, the supra- and infra- hyoid muscles participate in the movement of the tongue and the hyoid. As soon as the muscles EMG ceased, the tongue pressure disappeared, and the hyoid consequently started to retreat from the highest position in this study.

From the results of the interclass correlation coefficient, we noticed that the positive correlations between the EMG burst and hyoid activities, i.e., SH_on_, correlated well with the onset of slight/rapid movement of the hyoid, and IH_on_ correlated well with the onset of rapid movement of the hyoid. Based on the structural properties of the suprahyoid muscles and their potential for moving the hyoid^28,36^, the suprahyoid muscle provides the primary power to displace the hyoid in the anterior and superior directions. This may contribute to the high correlations between SH_on_ and T1 and T2. As for the close relationship between IH_on_ and T2, we speculated that the infrahyoid muscle may play a role in counterbalancing the suprahyoid muscle to stabilize the tongue and the hyoid during the swallowing process. However, no such case was observed for the SH_on_ or the TP_on_. This coincided with previous findings with surface electrodes and a midline disk-shaped pressure sensor showing that no significant correlation exists between the onset of the suprahyoid muscle EMG burst and the tongue tip touching the palate^4^. Interestingly, the absent time of swallowing-related muscles EMG positively correlated with not only the offset of tongue pressure at any site but also with the offset of the stationary phase of the hyoid. All the results suggest that there must be precise coordination in the motion between the tongue and the hyoid in healthy oropharyngeal swallowing^21^ because of the muscular connection. These interclass results suggest that we should be alert to any abnormal patterns in suprahyoid or infrahyoid muscles during swallowing because this might demonstrate dyskinesia of the tongue and the hyoid and vice versa.

Previous documents have reported that gender and age exert influences on swallowing behaviour^37,38^ and that the swallowing process would be affected by food properties and body positions^3,39^. Therefore, some limitations exist in the present study as the subjects recruited were all males with sitting in an upright position and swallowing just water bolus. Additionally, our present findings should be confirmed in the subjects with different swallowing behavior and compared with patients with swallowing problems. These issues during swallowing will be addressed in our future study design and performance.

In conclusion, the present results represent synchronous data from tongue pressure, muscle EMG and hyoid movement with non-invasive equipment that successfully exhibited the motor pattern and the temporal coordination of certain representative oropharyngeal events in healthy male subjects. The sensing system could be a good candidate for monitoring and evaluating the oropharyngeal process of swallowing and also has the potential to be useful for clinical work in dysphagia evaluation and rehabilitation.
